# Using the behavior change wheel (BCW) framework for health interventions focusing on diet and physical activity in the workplace: a scoping review

**DOI:** 10.3389/fpubh.2026.1890940

**Published:** 2026-08-20

**Authors:** Parastou Zerang, Daniel B. Scott, Sandra C. Dorman

**Affiliations:** 1 Laurentian University, Sudbury, ON, Canada; 2 The Center for Research in Occupational Safety and Health (CROSH), Laurentian University, Sudbury, ON, Canada

**Keywords:** COM-B model, health promotion, healthy eating, occupational health, physical activity, workplace intervention

## Abstract

**Background:**

The Behavior Change Wheel (BCW) offers a systematic approach for developing health promotion programs. Despite the importance of diet and physical activity programs in the workplace setting, the application of the BCW in developing these programs has not yet been examined. This scoping review aimed to identify and characterize workplace health promotion programs that target diet and physical activity, utilizing the BCW framework in their development.

**Methods:**

A systematic search was conducted across multiple databases in February 2024 and updated in January 2025 and further updated in June 2026. Studies were included if they reported workplace interventions targeting diet and/or physical activity behavior among workers using the BCW framework. Data on study characteristics, BCW implementation, identified Theoretical Domains Framework (TDF) domains, and Behavior Change Techniques (BCTs), as well as implementation outcomes, were extracted.

**Results:**

Thirteen studies, published across 34 papers, were identified, with the majority conducted in the UK and targeting office workers. Seven studies directly targeted diet and physical activity, while six addressed sedentary behaviors. Eight studies applied the BCW systematically and prospectively, four showed partial application, and one reflected nominal application. Knowledge was the most frequently identified TDF domain across all studies, and self-monitoring was the most frequently identified BCT. Eight studies progressed to pilot or feasibility trials ranging from 8 weeks to 12 months, with retention rates ranging from 69% to 100%, suggesting good acceptability and feasibility once participants were enrolled. However, intervention fidelity and sustainability were rarely reported, and alignment with the organization's mission was not reported in any study, limiting conclusions about the long-term and organizational success of these interventions.

**Conclusion:**

BCW provides structured guidance for developing health promotion programs in workplaces, with acceptable short-term retention rates among enrolled participants. However, future research needs to address its application in more diverse populations and workplace settings and give more attention to intervention fidelity, sustainability, and organizational alignment.

**Systematic review registration:**

https://osf.io/24hse, identifier: 24hse.

## Introduction

An unhealthy lifestyle significantly contributes to chronic diseases, both physically and mentally, which affect the quality of life and mortality (1). Physical Activity (PA) and diet are important modifiable lifestyle factors impacting chronic disease incidence (2). Because almost half of the world's population is workers who spend more than one-third of their days at the workplace, the workplace has enormous potential to affect the health of many people (3). Implementing Workplace Health Promotion Programs (WHPPs) may inspire employees to change their health behaviors and improve their health and wellbeing, leading to increased productivity (4, 5). Several WHPPs have been designed and implemented to improve PA and diet among workers (6–9). Evaluation of these interventions may provide meaningful insights into the interpretation of programs effectiveness by determining programs components as well as the facilitators and barriers influencing the implementation process (10, 11). However, the effectiveness of WHPPs varies depending on program content, study population, and intervention quality, highlighting the need for evidence-based, theory-informed approaches to intervention development (12–14).

Theoretical frameworks have increasingly informed the development and implementation of WHPPs (15–17). Several reviews indicate that theory-based interventions produce more statistically significant changes in health behavior than those that do not use theory (18–20). Theoretical behavior models and frameworks have been developed to understand behaviors; common models and theories include the Transtheoretical Model, the Social Cognitive Theory, the Theory of Planned Behavior, and the Behavior Change Wheel (BCW) (21, 22).

Evidence on the effectiveness of theory-informed workplace interventions is mixed. While some reviews suggest that theory-based interventions are more effective than those without a theoretical basis, others have found no significant difference (23–25).

**Figure 1:**
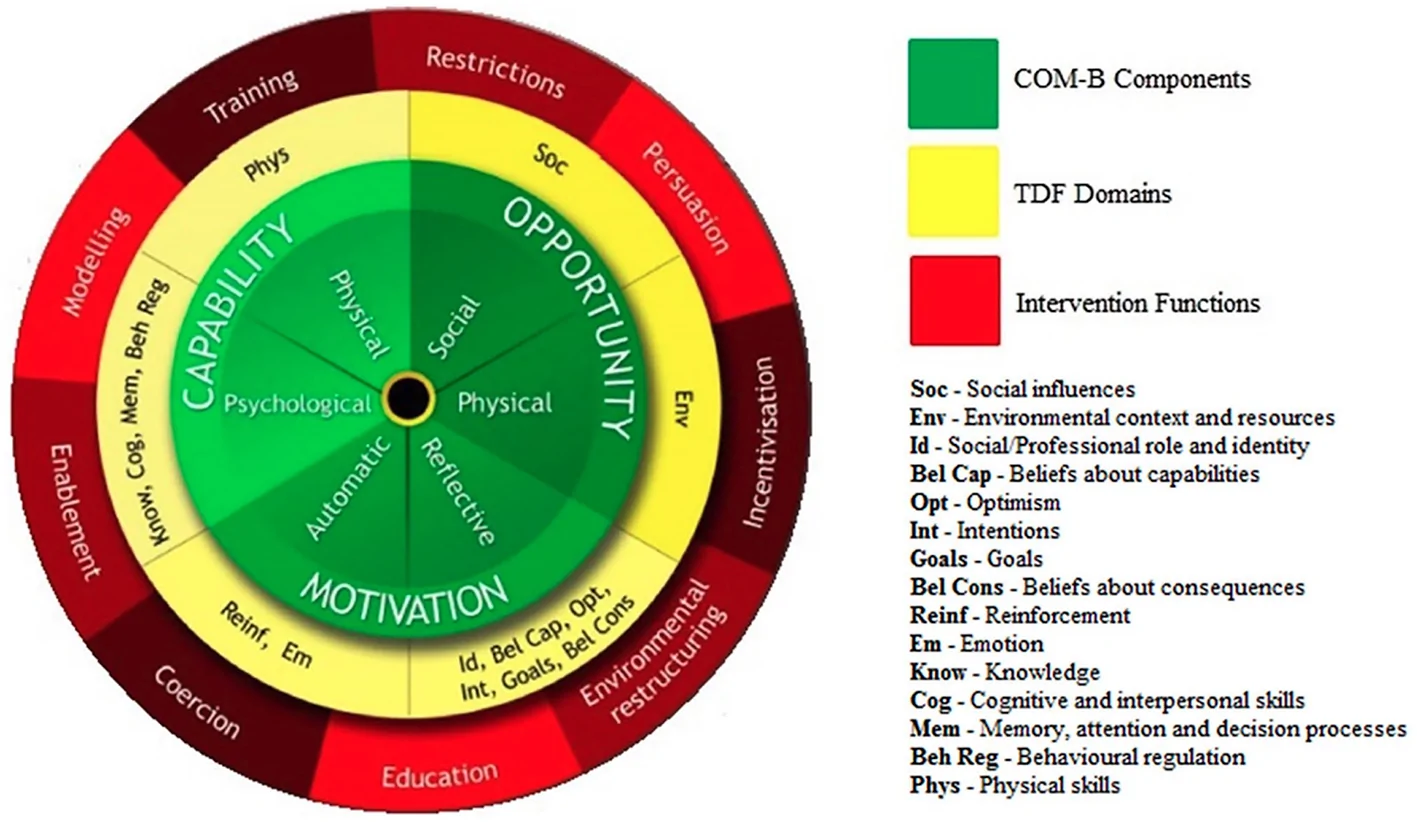
The Behavior Change Wheel; COM-B (green), TDF domains (yellow), and intervention functions (red) [reproduced from Powell and Thomas (72) under CC BY 4.0 license]; original framework from Michie et al. (22).

Among theoretical behavior models and frameworks, the BCW is one of the most comprehensive frameworks for systematically developing behavioral change interventions (Figure 1). Multiple-level factors, including individual, interpersonal and system, were considered during the development of the BCW (22). The BCW has been successfully used to develop workplace interventions and improve workers' health knowledge and behaviors (26, 27).

The BCW provides a systematic framework for informing the three stages of intervention development. In Stage 1, the COM-B model is used to understand behavior by identifying the Capability, Opportunity, and Motivation required for behavior change. The Theoretical Domains Framework (TDF) can then be applied to further explore barriers and facilitators across 14 theoretical domains. In Stage 2, intervention functions and policy categories are selected based on the COM-B/TDF analysis. In Stage 3, Behavior Change Techniques (BCTs) are identified as the active components of the intervention, linked to the selected intervention functions (22).

To date, no review has examined how the BCW has been applied to develop workplace health promotion interventions targeting diet and physical activity, or what has been achieved through this approach. Given the growing burden of lifestyle-related chronic diseases among working populations and the increasing adoption of BCW in intervention development, such a review is necessary to identify best practices and guide future research. As such, the purpose of this study was:

(1) To identify existing WHPPs that focus on PA and diet and apply the BCW for the development of an intervention.
(2) To identify how researchers implemented the steps of the BCW framework to promote health promotion interventions in the workplace (to identify best practices for using the BCW in this context).
(3) To identify the strengths and weaknesses of using the BCW to develop health promotion programs in the workplace.
(4) To determine the indicators of success or failure used.

## Method

This scoping review followed the Joanna Briggs Institute (JBI) Manual for Evidence Synthesis chapter on scoping reviews (28) and the Preferred Reporting Items for Systematic Reviews and Meta-Analyses Extension for Scoping Reviews (PRISMA-ScR) for reporting (29). In line with JBI guidance for scoping reviews, formal critical appraisal or risk-of-bias assessment of individual sources was not undertaken, as the aim was to map the extent and nature of the available evidence rather than to synthesize effect estimates (28). The review protocol was registered on osf.io on July 10th, 2024 (30).

### Inclusion /exclusion criteria

This scoping review includes articles reporting interventions developed for the workplace to change PA and/or diet behavior among workers using the BCW framework. Interventions targeting sedentary behavior reduction were also included as they represent an alternative approach to increasing PA (31). This allowed for a more comprehensive examination of BCW applications in WHPPs. As this review focused on workplace health interventions, participants included female and male employees aged 18 years or older, and the concept explored was interventions designed to change PA and diet among this population. Articles were included if the purpose of the intervention was to change the behaviors (diet and/or PA) using BCW. All types of studies were considered, and the type, content, duration, workplace setting, or country of origin of the intervention did not limit them. The context was the setting in which the intervention was delivered. Workplace interventions were included regardless of delivery format (e.g., face-to-face or online). Regarding evidence sources, eligible studies included those published in English, in peer-reviewed journals, conference papers, gray literature, popular literature, professional literature, and books. To be included, the identified studies were screened against the following eligibility criteria: (1) intervention studies using the Behavior Change Wheel (BCW), (2) providing a method and results section in the report, and (3) targeting behaviors related to doing PA and eating in the workplace. This scoping review considered papers involving the design or application of BCW in health promotion programs focusing on eating and PA behaviors at the workplace. However, studies were excluded if the research focused on using BCW combined with other theories or frameworks.

### Search strategy

The research team collaborated with a librarian to create a comprehensive search strategy. The databases searched were PubMed, CINAHL, Nursing and Allied Health (ProQuest), Evidence-based Medicine Multifile Reviews (OVID), Dissertations & Theses (ProQuest) and Google Scholar. These databases were searched in February 2024, updated in January 2025 and further updated in June 2026. The strategy was comprised of three main concepts: 1) Behavior Change Wheel (this concept appears in the literature with both UK and American spellings, with & without the “-al” suffix), 2) Health promotion in the workplace (the most precise MeSH terms are Occupational Health and Occupational Health Services), and 3) Eating and physical activity (relevant MeSH terms are Diet, Exercise, Physical Fitness, and Sedentary Behavior). The strategy was not limited to study type or year, and included, but was not limited to, dissertations, conference abstracts, pre-prints, and books. The entire search strategy is presented in Supplementary File 1.

### Source of evidence selection

**Figure 2:**
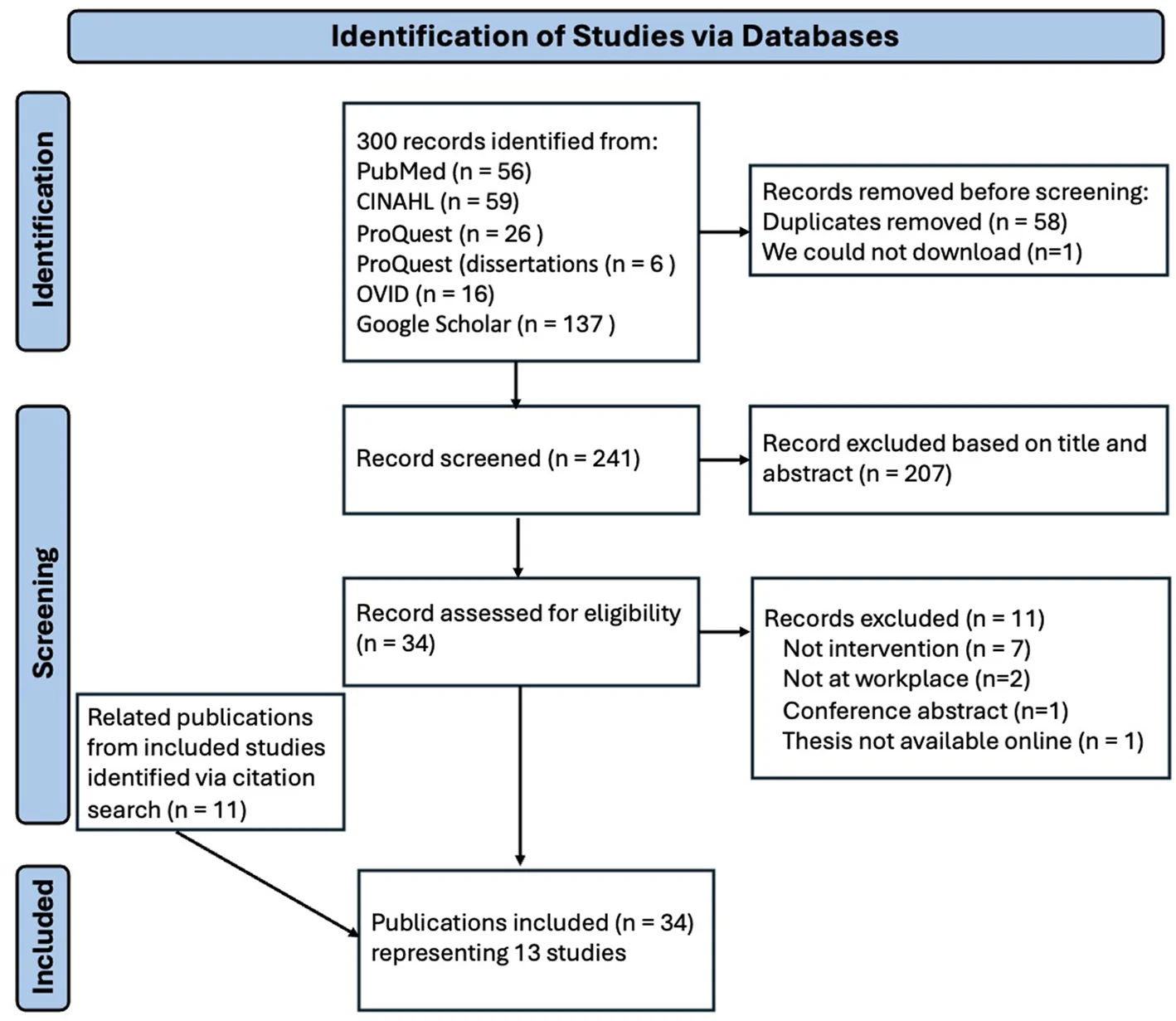
PRISMA-ScR flow diagram of study identification and selection process for the scoping review.

Studies identified in the database search were uploaded into JBI SUMARI for screening, inclusion, or exclusion from the review. After deduplicating the results, two reviewers independently screened titles and abstracts. These reviewers then met and, where a decision couldn't be made from the title and abstract alone, the reviewers screened the full texts of the selected articles. If consensus still couldn't be made, a third reviewer acted independently to resolve disagreements. Figure 2 illustrates the screening steps according to the PRISMA-ScR guidelines.

### Data extraction

The process of data extraction was divided into three categories.

(1) Intervention development stages: the process of intervention development involves multiple stages, including identifying what needs to be changed, designing, implementing, and evaluating the intervention. Each stage may lead to a separate publication. Therefore, to capture this comprehensive process, we considered each intervention as a single study comprising multiple related publications. In this part, all interventions with their related publications were identified.
(2) We extracted study characteristics from all identified publications related to the interventions: author(s) and year, country, target population, inclusion criteria, exclusion criteria, intervention setting, target behavior (s), method(s) of data collection (qualitative/quantitative), identified TDFs and BCTs, expected outcomes, measure of outcomes, and method(s) and type of delivery.
(3) When development of the intervention progressed to an actual or pilot study, we extracted implementation outcomes: sample size, retention rate, retention denominator basis and withdrawal/ missing data handling, characteristics of participants (compared with non-participants), project timeline, intervention fidelity (e.g., delivery adherence, completion rates), adaptations made during the implementation, and alignment with the organization's mission. Also, if the program was ongoing for more than 6 months, we extracted the results and the long-term adaptations (which elements were retained after the program was completed). Retention was defined as the number of participants who provided data or remained in the study at the final reported follow-up time point, divided by the number for whom the intervention began. For studies using cluster or individual randomization, this denominator was the number randomized; for single-arm or non-randomized designs, it was the number enrolled, as reported in the original publication. When a study reported retention at more than one time point, each time point was extracted and is presented separately in Supplementary File 5.

Selected articles and extracted data were reviewed multiple times to ensure that each component was accurately included or excluded as intended.

Included studies were also classified into three categories based on the direction and depth of their BCW application: first, systematic, prospective application (the study used COM-B and/or TDF before data collection to guide the identification of barriers and facilitators, selected intervention functions and BCTs based on this analysis, and completed all three BCW stages); second, partial application (the study identified barriers and facilitators without BCW guidance and mapped them onto COM-B and/or TDF afterwards, or did not complete all BCW stages); third, nominal application (the study referenced BCW as a guiding framework but did not report a behavioral analysis using COM-B and/or TDF).

## Results

### Search results

In total, the database search resulted in 300 records (Figure 2); one record was inaccessible and excluded. After removing duplicates (58 articles), title and abstract screening was conducted for 241 publications, and 34 of them proceeded to full-text screening. Based on defined inclusion and exclusion criteria, 23 publications were identified as relevant. Eleven additional publications were identified by searching the reference lists of the included publications; these were related publications from the same 13 studies already identified (for example, dissertations, protocols, or additional papers from the same research team) and were included to provide a complete picture of each study's development stages. In the final list, 34 publications were identified as eligible for inclusion in this scoping review (26, 27, 32–63). As described in Table 1 and supplementary file 2, these 34 publications represented 13 distinct intervention studies. Table 1 indicates, for each study, which publication addressed each stage of intervention development, and Supplementary File 2 summarizes the key content or outcomes reported in each of the 34 publications. Throughout this review, the term ‘study' refers to a research project that may include multiple related publications.

**Table 1:** Characteristics of included intervention studies and their related publications; this table presents the 13 intervention studies and their corresponding publications (*n* = 34). Each study comprised 1–5 publications describing different stages of the intervention development process. ✓ indicates the publication addressed that development stage; ✗ indicates it did not.

References	Year	Country	Dissertation	Data collection process	Intervention development process	Protocol for implementing pilot or actual interventions	Pilot or actual intervention implementation	Evaluation of the intervention	Total publications
Humphreys (63)	2026	UK	✗	✗	(63)	✗	✗	✗	1
Dicken (60), Niamh (61)	2024 and 2025	UK	✗	✗	(61)	(60)	✗	✗	2
Balter (34) and Halling Ullberg (62)	2024 and 2026	Sweden	✗	✗	✗	(34)	(62)	✗	2
Gibson (32, 33)	2024	UK	✗	(33)	(32)	✗	✗	✗	2
Hargreaves (35–37)	2020,2021 and 2023	New Zealand	✗	(35)	✗	(36)	(37)	✗	3
Huang (38–40)	2020 and 2023	UK	(38)	✗	(39)	✗	(40)	✗	3
Brierly (41–43)	2020, 2021 and 2022	UK	(41)	(42)	✗	✗	(43)	✗	3
Stephenson, (44–46)	2020 and 2021	UK	✗	(46)	(44)	✗	(45)	✗	3
Ojo (47–51)	2019 and 2022	UK	(47)	(48)	(49)	(50)	(51)	✗	5
Brogan, (26, 52, 53)	2020, 2021 and 2022	Australia	✗	(52)	✗	(53)	(26)	✗	3
Costello (59)	2018	UK	✗	✗	(59)	✗	✗	✗	1
Power, (27, 54)	2017 and 2021	UK	✗	(54)	(27)	✗	✗	✗	2
O'Connell, (55), Munir, (56), Edwardson, (57), Biddle, (58)	2015, 2018, 2018 and 2020	UK	✗	✗	(56)	(55)	(57)	(58)	4

### Study characteristics

Study characteristics are provided in Supplementary file 3. Most of the studies (10 out of 13) identified in this review were conducted in the UK (27, 32, 40, 43, 45, 51, 58–60, 63), and 8 out of 13 involved samples of office workers and took place in office settings (34, 36, 39, 43, 45, 49, 58, 63). In general, being an adult employee, working full-time with no physical disabilities that prevented participants from engaging in physical activity, were common inclusion criterion. In two studies, access to a smartphone during work hours was an additional inclusion criterion (34, 42). Employees were excluded if they planned to be absent for more than 2 weeks during the intervention, were currently participating in a health program, or were pregnant. Seven studies focused on diet and/or physical activity, with target populations of nurses, shift workers, office workers, working women in midlife, healthcare workers and a professional rugby league positional center (26, 27, 32, 34, 59, 60, 63). Six studies focused on sedentary behaviors at the workplace as target behaviors, with target populations of office workers across all studies (36, 39, 42, 46, 48, 56). Ten studies employed qualitative methods, including semi-structured interviews, focus groups and co-design workshops, to identify facilitators and barriers to the target behavior prior to intervention development (33, 35, 38, 42, 46, 48, 52, 54, 56, 63). Among these ten studies, seven used the COM-B model or TDF framework to guide the development of their data collection or analysis which were reported in a separate publication from the qualitative findings (32, 39, 42, 46, 48, 54, 56). In the remaining three studies, barriers and facilitators were identified without BCW guidance and were subsequently mapped onto the framework (35, 52, 63).

### Depth and direction of BCW application

The included studies varied in the direction and depth of their BCW application. Based on the classification criteria described in the Methods, three categories were identified (Supplementary File 4). Eight studies applied the BCW systematically and prospectively (27, 32, 39, 43, 44, 49, 56, 61). In these studies, COM-B and/or the TDF were used to guide the behavioral analysis of barriers and facilitators of behavior before intervention design, and all three stages of the BCW were completed.

Four studies showed a partial application (35, 52, 59, 63). In these studies, barriers and facilitators were identified without BCW guidance and were subsequently mapped onto the framework.

One study reflected a nominal application (34). This study reported a protocol for a future trial in which the BCW was referenced as a guiding framework, and BCTs were identified; however, a prior systematic behavioral analysis using COM-B or TDF was not reported.

Identified TDFs (Table 2) and BCTs (Table 3).

**Table 2:** Frequency of most commonly identified TDF domains.

TDF domain	Diet and/or PA (*n* = 5)	Sedentary (*n* = 5)	Total (*n* = 10)
Knowledge	5/5 (100%)	5/5 (100%)	10/10 (100%)
Environmental context and resources	4/5 (80%)	5/5 (100%)	9/10 (90%)
Beliefs about consequences	4/5 (80%)	5/5 (100%)	9/10 (90%)
Behavioral regulation	4/5 (80%)	4/5 (80%)	8/10 (80%)
Social influences	3/5 (60%)	5/5 (100%)	8/10 (80%)
Intentions	3/5 (60%)	4/5 (80%)	7/10 (70%)
Memory, attention and decision processes	2/5 (40%)	5/5 (100%)	7/10 (70%)
Beliefs about capabilities	3/5 (60%)	4/5 (80%)	7/10 (70%)
Goals	2/5 (40%)	4/5 (80%)	6/10 (60%)

**Table 3:** Frequency of most commonly identified BCTs.

BCT	Diet and/or PA (*n* = 6)	Sedentary (*n* = 5)	Total (*n* = 11)
Self-monitoring	6/6 (100%)	5/5 (100%)	11/11 (100%)
Information about health consequences	4/6 (67%)	5/5 (100%)	9/11 (82%)
Goal setting	4/6 (67%)	5/5 (100%)	9/11 (82%)
Social support	5/6 (83%)	4/5 (80%)	9/11 (82%)
Action planning	4/6 (67%)	5/5 (100%)	9/11 (82%)
Prompts and cues	3/6 (50%)	5/5 (100%)	8/11 (73%)
Feedback on behavior	2/6 (33%)	5/5 (100%)	7/11 (64%)

Across the 10 studies (out of 13) that reported TDFs (5 targeting diet and/or PA, and 5 targeting sedentary behavior), the most frequently identified TDF domain was knowledge, which was reported in all studies (10/10, 100%). Other commonly reported domains were environmental context and resources (9/10, 90%), beliefs about consequences (9/10, 90%), behavioral regulation (8/10, 80%), social influence (8/10, 80%), intentions (7/10, 70%), memory attention and decision processes (7/10, 70%), beliefs about capabilities (7/10, 70%), and goals (6/10, 60%). There were some differences between diet and/or PA interventions and sedentary behavior interventions. Social influences (5/5, 100%), memory, attention and decision processes (5/5, 100%), environmental context and resources (5/5, 100%), and beliefs about consequences (5/5, 100%) were more frequently reported in sedentary behavior studies than in diet and/or PA studies (3/5, 60%; 2/5, 40%; 4/5, 80%; 4/5, 80%, respectively).

Regarding BCTs, 11 (out of 13) studies reported BCTs (6 targeting diet and/or PA, and 5 targeting sedentary behavior). The most commonly reported BCTs were self-monitoring (11/11, 100%), goal setting (9/11, 82%), social support (9/11, 82%), action planning (9/11, 82%), and information about health consequences (9/11, 82%). In diet and/or PA studies, self-monitoring (6/6, 100%) and social support (5/6, 83%) were the most frequently reported BCTs. In sedentary behavior studies, self-monitoring (5/5, 100%), goal setting (5/5, 100%), action planning (5/5, 100%), information about health consequences (5/5, 100%), prompts and cues (5/5, 100%), and feedback on behavior (5/5, 100%) were reported in all studies. Detailed information on TDFs and BCTs identified in each study is provided in Supplementary File 3.

### Expected outcomes

Studies focusing on diet and/or PA (7 out of 13), had different expected outcomes compared to sedentary interventions. One study aimed to improve diet and PA among participants and to assess the feasibility of the intervention through a trial study (27, 54). One study expected improvements in health knowledge, dietary intake, and PA behavior, as measured by surveys and interviews to evaluate the changes and to find the experience of participants (26, 52, 53). One study targeting a rugby league player expected to find an increase in body mass (5.6 kg over 12 weeks) with improvement in anthropometric and strength assessment, examined by dual-energy X-ray absorptiometry scans and three-repetition maximums test (59). One study expected changes in dietary intake (nutrient consumption, climate-friendly and organic food) and increased active transportation, assessed using dietary scores and biomarkers (34, 62). One study aimed to reduce UPF intake and increase physical activity among NHS healthcare workers, with the feasibility and acceptability of the behavioral support program as primary outcomes (60, 61). One study aimed to co-design and test the acceptability of a digital exercise program for working women in midlife, using the Theoretical Framework of Acceptability as the primary outcome measure (63). The remaining study did not report expected outcomes (32).

Among the studies targeting sedentary behavior at work (6 out of 13), the expected outcomes included an increase in the mean number of opportunities to move and a reduction in sitting time, measured by app usage data and/or accelerometers. Additional expected outcomes across studies included feasibility and acceptability assessed using PRECIS-2 and RE-AIM frameworks and recruitment and retention logs (41–46), adherence and compliance assessed using app data and tracking (38–40), cardiometabolic risk factors measured by blood work (35–37, 41–43, 47–50), work-related factors including productivity, mood, job satisfaction, and work performance assessed using questionnaires (41–50, 55–58), and other health indicators including musculoskeletal problems, cognitive function, and quality of life assessed using questionnaires (55–58).

Interventions were delivered through various methods, including educational workshops and seminars, coaching consultations, printed materials (leaflets), smartphone apps, email and SMS, logbooks, electronic prompts, team competitions, workplace champions, and the provision of height-adjustable desks, pedometers, and accelerometers. More details on outcomes and measurement methods, and types of delivery are provided in Supplementary File 3.

### Implementation of the intervention

Across all 13 studies, 8 conducted pilot or full trials to assess the feasibility of interventions (26, 37, 40, 43, 45, 51, 57, 62), one was a single-case study that developed and delivered the intervention with one participant but did not undertake a pilot or feasibility trial (59), and four did not progress to pilot or trial stages (33, 54, 60, 63). Among 7 studies targeting diet and PA, one study conducted a pilot and feasibility study with 37 office workers over 8 weeks. The retention rate was 89% (33 of 37) at the end of the 8-week intervention. Workshop attendance was acceptable (80% and 78% for the two intervention arms), and results showed a significant reduction in diet-related CO_2_e and sedentary time in the sustainable lifestyle arm (62). One study was a 12 week intervention for a rugby team player to gain weight and improve anthropometric and strength assessment; including changes in body mass (6.2 kg increase) with improvements in diet quality, strength, and body composition (59). One study conducted a complete feasibility study with a sample size of 99 (graduate nurses) for 6 months. In this study, the retention rate was 69% (68 of 99) at 6 months, with 52% (52 of 99) completing all three surveys (at baseline, 3 months and 6 months). No significant differences were reported between participants and non-participants. The authors did not mention any adaptations to the intervention or alignment to the organization's mission during the study. After 6 months, participants demonstrated improved health knowledge, correctly identifying the recommended intakes of fruits, vegetables, and physical activity. Fruit consumption increased, while vegetable intake showed minimal change. Although takeaway food consumption declined, the intake of some discretionary foods increased. Across all three time points, engagement in leisure-time physical activity remained low (26). Four studies did not include pilot or trial stages (33, 54, 60, 63).

All six studies targeting sedentary behavior were followed by a full or pilot trial to assess the feasibility. One study with 58 university staff over 24 weeks had a retention rates of 89% (52 of 58) at week 12 and 81% (43 of 53, excluding four participants who left employment during the study) at week 24. Participants self-reported taking 62%−69% of opportunities to move during the intervention period. No significant change in the number of opportunities to move was observed, but moderate-to-vigorous physical activity at work, self-assessed work productivity, and musculoskeletal health (reduced pain reporting from 98% to 85%) were observed. Weight and waist circumference were improved at follow-up, while other cardiometabolic markers showed no significant changes. No improvements in psychological wellbeing were found (37). One study with 15 sedentary office workers over 8 weeks reported the retention rate of 100% (15 of 15) and 83% adherence to the tracking protocol (mean 25 of 30 workdays tracked). No significant changes in occupational sitting or physical activity behaviors observed. However, significant improvements were found in psychosocial outcomes, including automaticity of microbreak behaviors, retrospective memory of breaks, and prospective memory of breaks. No changes in work fatigue were observed (40). One study with 24 police officers over 8 weeks showed 79% retention rate (19 of 24). Low fidelity to the protocol for the intervention, with one adaptation (personalized messages added in weeks one and five) were reported. Results showed significant improvements in workplace sitting and standing, with a reduction in sitting time by 17.65 min per workday and an increase in standing time by 15.49 min. Prolonged sitting bouts decreased significantly. Small improvements were observed in body weight (0.86 kg reduction) and positive affect. No significant changes were observed in other cardiometabolic markers, wellbeing, stress, or job performance (43). One study with 56 office workers across three companies over 8 weeks reported different participation rates (19%, 38%, and 23% of eligible employees in each company) and retention rates (95% (19 of 20), 90% (18 of 20), and 81% (13 of 16) in each group). All intervention components were delivered; however, 24% of participants did not receive educational elements, and 26%−44% reported technical issues. The study concluded that the mobile app-based intervention was feasible to implement in the workplace, with moderate to low participant satisfaction (45). One study with 44 desk-based employees over 8 weeks reported 84% participation among contacted employees and a retention rate of 91% (40 of 44 completed). Results showed an 11% reduction in workplace sitting time (from 76% to 60% of work hours), replaced predominantly by standing (an 11% increase). Participants reported significantly increased engagement, motivation, and productivity while sitting. No significant changes were found in stress, mood, wellbeing, or cardiometabolic biomarkers (51). One study with 146 NHS office staff (77 intervention, 69 control) across 37 clusters over 12 months reported retention rates of 83% (121 of 146) at 3 months, 79% (115 of 146) at 6 months, and 75% (109 of 146) at 12 months. Results showed a successful reduction in occupational sitting time of 83 minutes per workday at 12 months, which was maintained in the short-, medium-, and long-term. Significant improvements were observed in work-related outcomes, including work engagement, job performance, recovery from fatigue, and sickness presenteeism. Improvements were also observed in cognitive function (reaction times) and quality of life. Some improvements in musculoskeletal outcomes were noted. No notable changes were found in job satisfaction or sickness absence (57).

More details on implementation outcomes are provided in Supplementary file 5.

### Findings by stage of study completion

The findings reported above came from studies at different stages of completion. To allow each finding to be read within its methodological context, rather than treating all studies as equivalent, the studies were grouped into three stages: formative/development, pilot/feasibility, and full-scale implementation (Supplementary File 6). The range of findings that could be reported widened across these stages: formative/development studies reported the behavioral analysis and intervention design; pilot and feasibility studies additionally reported retention and preliminary behavioral outcomes; and only the single full-scale study reported definitive effectiveness with long-term follow-up.

## Discussion

This scoping review provides the first examination of WHPPs focusing on diet and PA that used the BCW framework in their development.

The identification of 13 studies across 34 papers shows a gradual increase in the adoption of the BCW framework for workplace health promotion interventions targeting diet and physical activity following its introduction in 2014 (22). The concentration of studies in the UK (10 out of 13) may reflect the framework's origin and early adoption in British research contexts. However, this concentration also means the evidence base may not reflect the diversity of workplace cultures in other countries, a point to consider when applying these findings elsewhere. Additionally, the growing application of BCW in workplace interventions reflects its increasing recognition as a tool for developing systematic interventions. Among all studies, seven focused directly on diet and/or PA; we also included six studies focused on reducing sitting time as a way to increase PA, which indicates that researchers using BCW to target both direct health behavior and related behavior that are easier to change in the workplace. Most studies (8 out of 13) focused on office workers who sit for extended periods and often have poor eating habits; among them, six studies targeted prolonged sitting (35, 38, 42, 44, 49, 55) at the workplace, and two studies targeted diet and PA (34, 63). The first was initially a protocol for a randomized controlled trial that progressed to a pilot study (34, 62). Since office workers represent a significant portion of the workforce, a strong understanding of how BCW can be applied in this setting to change diet and PA behavior is valuable.

### Implementing the steps of the BCW

Across the included studies, BCW was implemented in different ways. Among them, ten studies used interviews, focus groups or co-design workshops to determine the barriers and facilitators of the target behavior before intervention design (33, 35, 38, 42, 46, 48, 52, 54, 56, 63). One study used a systematic literature review with one-to-one participant sessions guided by COM-B and TDF to identify barriers and facilitators (60); one study was developed without prior identification of barriers and facilitators (34); and one study used clinical case assessment and COM-B analysis to identify barriers within a specific clinical context (59). Using diverse methods to identify barriers and facilitators is a strength of the framework, as it allows researchers to adapt BCW to different populations, settings, and resources. However, this flexibility also leads to variation in how systematically the framework is applied, making it harder to compare studies and assess the rigor of each application. In addition, all studies identified TDFs and/or BCTs. The most commonly identified TDF for diet and PA studies was knowledge (100%). Reviews of studies that identified barriers and facilitators of PA and diet by using BCW are limited; however, our findings were partially aligned with a systematic review by Spiteri et al. (64), who found environmental context and resources as the most frequent TDF for PA among middle-aged and older adults. In our review of diet and/or PA studies, environmental context and resources was also frequently identified (4/5, 80%), ranking second after knowledge. The differences primarily may have stemmed from not analyzing the studies that applied BCW instructions; instead, they mapped their results into TDF. The other reason could be the study's population age group. Regardless of the method used to collect data on barriers and facilitators, the consistent identification of knowledge as a barrier across all diet/PA studies is notable; even in workplace populations that may be assumed to have basic health knowledge, gaps remain in specific areas such as guidelines, health impacts, and practical application. This suggests that interventions should provide targeted, context-specific knowledge rather than assuming general awareness is sufficient. In studies targeting diet and/or PA, the most identified BCTs were self-monitoring (100%) and social support (83%); for studies focusing on sitting behavior, self-monitoring (100%), goal setting (100%), action planning (100%), information about health consequences (100%), prompts and cues (100%), and feedback on behavior (100%) were the most identified BCTs. These findings align with the meta-regression results by Michie et al. (65), which revealed that interventions using self-monitoring with at least one other technique were significantly more effective than other interventions on healthy eating and PA.

While the BCW informed each of the included interventions, the depth and direction of its application varied considerably, with eight of the thirteen studies applying it systematically and prospectively. This distinction matters because the BCW is designed as a systematic process in which intervention functions and BCTs are meant to follow from a COM-B/TDF-based behavioral analysis. In the four studies where this analysis was conducted retrospectively (35, 52, 59, 63), the resulting BCTs may therefore be less closely tailored to the barriers and facilitators of the populations they were intended for.

Beyond the depth and direction of BCW application, the studies also differed in how far they progressed. Organizing the findings by stage of study completion helps to read each finding within its methodological context, rather than treating all studies as equivalent (Supplementary File 6). It also made visible a pattern in how far different interventions progressed: BCW-informed interventions on sedentary behavior in the workplace appear more advanced in implementation terms, having reached pilot trials in all cases and one full-scale trial, while interventions on diet and physical activity, although showing progress with two pilot studies completed (26, 62), remain at an earlier stage and have not yet reached definitive evaluation. This points to diet and physical activity at work as areas where BCW-informed interventions remain limited and are in their early stages. It should be noted that, within the BCW, the TDF domains and BCTs are identified during the early development stages and do not depend on whether the intervention was later piloted or implemented; for this reason, they were reported in Tables 2, 3 rather than grouped by stage.

### Strengths and weaknesses of using BCW

Using BCW in the development of WHPPs has several strengths and some weaknesses. Regarding strengths, it provided a straightforward, systematic, step-by-step approach from understanding behavior to designing interventions, which allowed the researchers to address individual, social, and environmental factors simultaneously. In this framework, understanding capability, opportunity, and motivation (COM-B) and using TDFs to identify specific barriers and facilitators in the workplace helped to ultimately determine the most effective BCTs (as active components of intervention) to change the target behavior among employees. This systematic approach resulted in high retention rates among enrolled participants (ranging from 69% to 100%), suggesting good intervention acceptability and feasibility. Regarding weaknesses, the challenge of small, self-selected samples, which limits generalizability, was highlighted in the reviewed papers, especially when data were extracted from single worksites or specific occupational groups (26, 35, 40, 42, 43, 45, 50, 54, 62, 63). These limitations have been reported in other workplace health interventions (66, 67). In applying BCW, one limitation was not using COM-B and TDF to guide the interview questions; three studies conducted the interview first and then mapped it into TDF (a departure from the intended systematic approach) (35, 52, 63). Similarly, one study used clinical case assessment (59) and then mapped findings onto COM-B and TDF. Additionally, Power et al. (54) highlighted two other limitations: first, proposed interventions may identify all available intervention functions and policy categories, which may suggest a lack of sufficient specificity in the BCW framework to guide precise intervention development. Second, the framework's replicability in identifying the most appropriate BCTs and implementing them is subjective, as different research teams may identify different BCTs and employ different implementation methods.

A further limitation concerns the reporting of TDF domains and BCTs, which were highly heterogeneous across the included studies. Some studies provided extensive lists of domains and techniques, whereas others reported only a few or none (see Supplementary file 3). The frequency patterns in this review should therefore be interpreted with caution. They may partly reflect how fully each study reported its analysis, rather than real differences in the domains and techniques behind the interventions. Future studies using the BCW should report the identified TDF domains and BCTs more fully and consistently, so that interventions can be compared more reliably.

### Indicators of success or failure

Studies employed both implementation outcomes and behavioral outcomes to measure success and failure. Implementation outcomes varied across studies.

As demonstrated by Proctor et al. (68), implementation outcomes provide a comprehensive framework for evaluating intervention success, which contains eight distinct elements: acceptability, adoption, appropriateness, feasibility, fidelity, implementation cost, penetration, and sustainability. In the reviewed studies, implementation success was primarily evaluated by retention rates as a measure of feasibility (26, 37, 40, 43, 45, 51, 57, 62) and by participant feedback (26, 37, 40), including interview data and app usage data, as a measure of acceptability. One study additionally used the Theoretical Framework of Acceptability to assess the acceptability of a digital exercise program (63). Retention rates reported among the eight studies that conducted pilot or feasibility trials ranged from 69% to 100%, indicating strong participant engagement once enrolled (Because studies reported retention at different time points and in different formats, the rates are presented as percentages, separately for each study and time point, in Supplementary File 5). However, intervention fidelity, which ensures interventions are delivered as planned, was rarely reported; only one study reported (low) delivery fidelity to protocol (43), which limits conclusions about the quality of intervention delivery. Two studies assessed implementation using established frameworks, including PRECIS-2 and RE-AIM (43, 45), while three studies used interviews and app usage data to evaluate feasibility and acceptability (26, 37, 40). Notably, appropriateness and implementation cost were not reported in any of the reviewed studies, whereas adoption (43), penetration (43), and sustainability (40, 43) were rarely reported, representing important gaps in evaluating the broader success and long-term reach of BCW-informed workplace interventions.

These gaps are important in the workplace setting. Sustainability was assessed in only two studies, but a workplace intervention can have a lasting effect only if it continues beyond the project and becomes part of the routine. In these studies, the findings varied: in one study, most participants kept using the intervention on their own for a while after it ended (40), and in another, the organization intended to keep part of the intervention as routine practice (43).

Alignment with the organization's mission is considered important for whether workplace interventions are adopted and sustained. None of the 13 included studies formally assessed or reported this variable, and this absence should be read as a reporting gap rather than evidence that the interventions were poorly aligned with their organizations. Two studies described organizational engagement that may reflect alignment, even though they did not measure it directly: in SMArT Work, senior management publicly supported the study through regular staff communications (57), and in A-REST, feedback from an organizational health board suggested interest in keeping parts of the intervention as routine practice (43). It is not clear, though, whether these examples reflect real organizational alignment or simply informal, individual-level support. Future studies should use validated tools to assess organizational mission alignment directly and evaluate its role in adoption and sustainability separately from participant-level feasibility outcomes.

Behavioral outcome selection was also consistent with established frameworks for workplace health interventions. Sedentary behavior interventions appropriately used sitting time as the primary outcome, completed by work-related outcomes (productivity, work engagement, job performance) and health indicators (69, 70). Diet and PA interventions measured dietary intake, physical activity levels, and weight-related outcomes, which align with recommended measures for workplace health interventions (7, 71). One study additionally measured diet-related carbon footprint as a novel indicator of sustainable food habits (62). In this review, sedentary behavior interventions measured success primarily through reductions in sitting time, with additional outcomes including improvements in work-related outcomes (productivity, work engagement, job performance) and health indicators (cardiometabolic markers, musculoskeletal health, quality of life) (37, 43, 57). Studies targeting diet and PA assessed improvements in health knowledge, fruit and vegetable intake, and PA levels (53, 59) and reductions in dietary carbon footprint and sedentary time (62). Only one study delivered and evaluated the intervention over 12 months (57); the shorter durations in the remaining studies limit conclusions about whether behavior change was maintained.

Together, these findings suggest that BCW provides a viable strategy for developing effective workplace health interventions. However, the evidence base remains limited: only one study (SMArT Work) reached the level of definitive effectiveness, and further trials, particularly for diet and PA, are needed to establish whether BCW-informed interventions can produce sustained behavior change across diverse workplace settings. The impact of BCW-informed workplace interventions goes beyond individual behavior change. Improvements in productivity, work engagement, and reduced sickness presenteeism (37, 51, 57) suggest that these interventions can benefit both employees and employers, making them more likely to be supported by organizations.

### Best practices for using the BCW

Regarding the implementation of BCW in the reviewed studies and the identified limitations, several recommendations emerge for future research:

(1) Studies should use COM-B and TDF to guide data collection from the beginning, rather than collecting data first and then mapping it to the framework later.
(2) Researchers should aim to collect data from multiple sources and employ a variety of methods to collect data on barriers and facilitators of the target behavior prior to intervention design. While ten studies employed qualitative methods, to identify facilitators and barriers to the target behavior prior to intervention development (33, 35, 38, 42, 46, 48, 52, 54, 56, 63), Michie et al. (22) explicitly recommend collecting data from as many relevant sources as possible using a variety of methods including interviews, focus groups, questionnaires, direct observation, and review of relevant documents, noting that triangulating data from multiple sources and methods strengthens understanding of the target behavior.
(3) Studies should include more diverse workplace populations. The reviewed studies show a pattern in which sedentary behavior interventions were predominantly conducted in office-based settings, whereas diet and physical activity interventions were mostly conducted in healthcare settings. Although two recent studies have begun to address this gap by targeting office workers (62) and working women in university and private sector settings (63), both remain in their early stages of development and have not yet been evaluated for effectiveness. As such, further research in diverse non-healthcare workplace settings remains needed to improve the generalizability of current evidence to diverse occupational groups.
(4) It is noted that the proposed interventions may identify all available intervention functions and policy categories, suggesting insufficient specificity in the BCW framework to guide precise intervention development. To address this, studies should apply more explicit criteria when selecting intervention functions and policy categories to ensure more targeted and replicable intervention development.
(5) To address key limitations in study design, future research should use larger samples with a control group whenever possible and plan for a longer intervention period, including both implementation outcomes (e.g., intervention fidelity) and long-term outcomes to assess sustainability.

While this review suggests that BCW could provide a valuable framework for the systematic development of interventions, further research should address the gap in the long-term application of these interventions and their implementation across different types of workplaces.

## Conclusion

This study provided the first review of the application of BCW in developing WHPPs focused on PA and diet. It emphasized the significant potential of applying this framework in this field. Although BCW offers practitioners structured guidance for health program development, addressing current gaps related to population diversity and long-term maintenance remains essential for maximizing its effectiveness in workplace health promotion.

## Data Availability

The original contributions presented in the study are included in the article/Supplementary material, further inquiries can be directed to the corresponding author.

## Supplementary material

Supplementary File 1 Search strategy.

Supplementary File 2 Summary of key content and outcomes reported in each of the 34 included publications.

Supplementary File 3 Study characteristics.

Supplementary File 4 BCW classification (direction and depth of application).

Supplementary File 5 Implementation outcomes.

Supplementary File 6 Grouping of studies by stage of completion.
