# Supplementary material for: Using the behavior change wheel (BCW) framework for health interventions focusing on diet and physical activity in the workplace: a scoping review
Source: Front Public Health. 2026 Aug 20;14:1890940. doi: 10.3389/fpubh.2026.1890940 (PMC13538869; doi:10.3389/fpubh.2026.1890940)
Supplement: Supplementary file 1 [file Table_1.docx]

Search strategy: BCW workplace interventions targeting eating and physical activity

# Overview

From the draft study protocol:

The identified studies will be screened against the following eligibility criteria: (1) intervention studies using the Behavioural Change Wheel (BCW), (2) providing a detailed method and results section in the report, and (3) targeting behaviours related to eating and doing physical activity in the workplace.

## Major concepts

- Behavioural Change Wheel (BCW)
- Health promotion in the workplace
- Eating and physical activity

# PubMed

Keyword searches will be restricted to the Title/Abstract for precision.

Search results for individual aspects have been updated as of 2024-02-02.

## Concept 1: Behavioural Change Wheel

This concept appears in the literature with both UK and American spellings, with & without the "-al" suffix.

No MeSH term serves as a direct equivalent.

"behavior change wheel"[Title/Abstract] OR "behaviour change wheel"[Title/Abstract] OR "behavioral change wheel"[Title/Abstract] OR "behavioural change wheel"[Title/Abstract] OR "COM-B"[Title/Abstract]

1,410 results.

With so few results, this concept is going to be the limiting factor.

## Concept 2: Health promotion in the workplace

The most precise MeSH terms are **Occupational Health** and **Occupational Health Services**.

"Occupational Health"[MeSH] OR "Occupational Health Services"[MeSH]

46,455 results.

Adding a keyword search to expand the results:

"Occupational Health"[MeSH] OR "Occupational Health Services"[MeSH] OR "occupational health"[Title/Abstract]

58,934 results.

To further broaden the search, we include the intersection of the concepts of "health promotion" and "workplace".

### Health promotion

The MeSH term "Health Promotion" returns 85,658 results.

Expanding this with some keyword searches for common synonyms:

"Health Promotion"[MeSH] OR "wellness program"[Title/Abstract] OR "health promotion"[Title/Abstract] OR "behavior change"[Title/Abstract] OR "behaviour change"[Title/Abstract]

128,555 results.

### Workplace

The **Workplace** MeSH term is the most accurate. However, keyword searches for synonyms for workplace, as well as a search for employee, will provide the appropriate breadth:

"Workplace"[MeSH] OR "Employee*"[Title/Abstract] OR "worker*"[Title/Abstract] OR "work environment"[Title/Abstract] OR "workplace*"[Title/Abstract] OR "work place*"[Title/Abstract] OR "workspace*"[Title/Abstract] OR "work space*"[Title/Abstract]

OR "office*"[Title/Abstract] OR "desk-based"[Title/Abstract]

434,219 results.

### Intersection of Health Promotion & Workplace

The intersection of the health promotion and workplace concepts is 12,482 results:

("Workplace"[MeSH] OR "Employee*"[Title/Abstract] OR "worker*"[Title/Abstract] OR "work environment"[Title/Abstract] OR "workplace*"[Title/Abstract] OR "work place*"[Title/Abstract] OR "workspace*"[Title/Abstract] OR "work space*"[Title/Abstract]

OR "office*"[Title/Abstract] OR "desk-based"[Title/Abstract]) AND ("Health Promotion"[MeSH] OR "wellness program"[Title/Abstract] OR "health promotion"[Title/Abstract] OR "behavior change"[Title/Abstract] OR "behaviour change"[Title/Abstract])

Added to the Occupational Health search, we have:

(("Workplace"[MeSH] OR "Employee*"[Title/Abstract] OR "worker*"[Title/Abstract] OR "work environment"[Title/Abstract] OR "workplace*"[Title/Abstract] OR "work place*"[Title/Abstract] OR "workspace*"[Title/Abstract] OR "work space*"[Title/Abstract]

OR "office*"[Title/Abstract] OR "desk-based"[Title/Abstract) AND ("Health Promotion"[MeSH] OR "wellness program"[Title/Abstract] OR "health promotion"[Title/Abstract] OR "behavior change"[Title/Abstract] OR "behaviour change"[Title/Abstract])) OR ("Occupational Health"[MeSH] OR "occupational health"[Title/Abstract] OR "Occupational Health Services"[Mesh])

67,736 results

## Concept 3: Eating and physical activity

Relevant MeSH terms are **Diet**, **Exercise**, **Physical Fitness,** and **Sedentary Behavior**.

We also include the keywords "diet*", "eating", "exercise*", “fitness” and "physical activity":

"Diet"[MeSH] OR "Exercise"[MeSH] OR "Physical Fitness"[MeSH] OR "Sedentary Behavior"[MeSH] OR "exercise*"[Title/Abstract] OR "physical activity"[Title/Abstract] OR "diet*"[Title/Abstract] OR "eating"[Title/Abstract] OR "fitness"[Title/Abstract] OR "sedentary"[Title/Abstract]

1,336,624 results.

## Consolidated search

Adding concept 2 and concept 3 gives us:

((("Workplace"[MeSH] OR "Employee*"[Title/Abstract] OR "worker*"[Title/Abstract] OR "work environment"[Title/Abstract] OR "workplace*"[Title/Abstract] OR "work place*"[Title/Abstract] OR "workspace*"[Title/Abstract] OR "work space*"[Title/Abstract]

OR "office*"[Title/Abstract] OR "desk-based"[Title/Abstract) AND ("Health Promotion"[MeSH] OR "wellness program"[Title/Abstract] OR "health promotion"[Title/Abstract] OR "behavior change"[Title/Abstract] OR "behaviour change"[Title/Abstract])) OR ("Occupational Health"[MeSH] OR "occupational health"[Title/Abstract] OR "Occupational Health Services"[Mesh])) AND ("Diet"[MeSH] OR "Exercise"[MeSH] OR "Physical Fitness"[MeSH] OR "Sedentary Behavior"[MeSH] OR "exercise*"[Title/Abstract] OR "physical activity"[Title/Abstract] OR "diet*"[Title/Abstract] OR "eating"[Title/Abstract] OR "fitness"[Title/Abstract] OR "sedentary"[Title/Abstract]])

5,246 results.

Adding the BCW concept gives [42 results](https://pubmed.ncbi.nlm.nih.gov/?term=%28++%28+++%28++++%28+%09%28%22Workplace%22%5BMeSH%5D+OR+%22Employee*%22%5BTitle%2FAbstract%5D+OR+%22worker*%22%5BTitle%2FAbstract%5D+OR+%22work+environment%22%5BTitle%2FAbstract%5D+OR+%22workplace*%22%5BTitle%2FAbstract%5D+OR+%22work+place*%22%5BTitle%2FAbstract%5D+OR+%22workspace*%22%5BTitle%2FAbstract%5D+OR+%22work+space*%22%5BTitle%2FAbstract%5D+OR+%22office*%22%5BTitle%2FAbstract%5D+OR+%22desk-based%22%5BTitle%2FAbstract+%09%29+%09AND+%09%28%22Health+Promotion%22%5BMeSH%5D+OR+%22wellness+program%22%5BTitle%2FAbstract%5D+OR+%22health+promotion%22%5BTitle%2FAbstract%5D+OR+%22behavior+change%22%5BTitle%2FAbstract%5D+OR+%22behaviour+change%22%5BTitle%2FAbstract%5D+%09%29++++%29++++OR+%28%22Occupational+Health%22%5BMeSH%5D+OR+%22occupational+health%22%5BTitle%2FAbstract%5D+OR+%22Occupational+Health+Services%22%5BMesh%5D++++%29+++%29++%29++AND+%28%22Diet%22%5BMeSH%5D+OR+%22Exercise%22%5BMeSH%5D+OR+%22Physical+Fitness%22%5BMeSH%5D+OR+%22Sedentary+Behavior%22%5BMeSH%5D++OR+%22exercise*%22%5BTitle%2FAbstract%5D+OR+%22physical+activity%22%5BTitle%2FAbstract%5D+OR+%22diet*%22%5BTitle%2FAbstract%5D+OR+%22eating%22%5BTitle%2FAbstract%5D+OR+%22fitness%22%5BTitle%2FAbstract%5D+OR+%22sedentary%22%5BTitle%2FAbstract%5D++%29+%29+AND+%28%28%22behavior+change+wheel%22%5BTitle%2FAbstract%5D+OR+%22behaviour+change+wheel%22%5BTitle%2FAbstract%5D+OR+%22behavioral+change+wheel%22%5BTitle%2FAbstract%5D+OR+%22behavioural+change+wheel%22%5BTitle%2FAbstract%5D+OR+%22COM-B%22%29%29) as of 2025-01-24:

(

(

(

(

("Workplace"[MeSH] OR "Employee*"[Title/Abstract] OR "worker*"[Title/Abstract] OR "work environment"[Title/Abstract] OR "workplace*"[Title/Abstract] OR "work place*"[Title/Abstract] OR "workspace*"[Title/Abstract] OR "work space*"[Title/Abstract] OR "office*"[Title/Abstract] OR "desk-based"[Title/Abstract

)

AND

("Health Promotion"[MeSH] OR "wellness program"[Title/Abstract] OR "health promotion"[Title/Abstract] OR "behavior change"[Title/Abstract] OR "behaviour change"[Title/Abstract]

)

)

OR ("Occupational Health"[MeSH] OR "occupational health"[Title/Abstract] OR "Occupational Health Services"[Mesh]

)

)

)

AND ("Diet"[MeSH] OR "Exercise"[MeSH] OR "Physical Fitness"[MeSH] OR "Sedentary Behavior"[MeSH] OR "exercise*"[Title/Abstract] OR "physical activity"[Title/Abstract] OR "diet*"[Title/Abstract] OR "eating"[Title/Abstract] OR "fitness"[Title/Abstract] OR "sedentary"[Title/Abstract]

)

)

AND (("behavior change wheel"[Title/Abstract] OR "behaviour change wheel"[Title/Abstract] OR "behavioral change wheel"[Title/Abstract] OR "behavioural change wheel"[Title/Abstract] OR "COM-B"))

# Evidence Based Medicine Reviews Multifile

Translating the PubMed search to Evidence Based Medicine Reviews Multifile (on the Ovid platform):

EBM Reviews - Cochrane Database of Systematic Reviews <2005 to January 22, 2025>

EBM Reviews - ACP Journal Club <1991 to January 2025>

EBM Reviews - Database of Abstracts of Reviews of Effects <1st Quarter 2016>

EBM Reviews - Cochrane Clinical Answers <January 2025>

EBM Reviews - Cochrane Central Register of Controlled Trials <December 2024>

EBM Reviews - Cochrane Methodology Register <3rd Quarter 2012>

EBM Reviews - Health Technology Assessment <4th Quarter 2016>

EBM Reviews - NHS Economic Evaluation Database <1st Quarter 2016>

1	("Workplace" or "Employee*" or "worker*" or "office*" or "desk-based").ab,ct,hw,kw,sh,ti.	35407

2	("Health Promotion" or "wellness program" or "health promotion" or "behavior change" or "behaviour change").ab,ct,hw,kw,sh,ti.	23191

3	1 and 2	2538

4	"Occupational Health".ab,ct,hw,kw,sh,ti.	2469

5	3 or 4	4457

6	("Diet" or "Exercise" or "Sedentary Behavior" or "diet*" or "eating" or "physical activity" or "fitness").ab,ct,hw,kw,sh,ti.	275190

7	5 and 6	1656

8	("behavior* change wheel" or "behaviour* change wheel" or "COM-B").ab,ct,hw,kw,sh,ti.	299

9	7 and 8	11

11 results as of 2025-01-24 (up from 8 results).

# CINAHL

Searches are performed with **Apply related words** and **Apply equivalent subjects** disabled.

## Concept 1: Behavioural Change Wheel

TI ( "behavior* change wheel" or "behaviour* change wheel" or "COM-B" ) OR AB ( "behavior* change wheel" or "behaviour* change wheel" OR "COM-B" )

660 results

## Concept 2: Health promotion in the workplace

### Occupational health

TI ("Occupational Health") OR AB ("Occupational Health") OR MW("Occupational Health")

41,940 results

### Health promotion

TI ("Health Promotion" or "wellness program" or "health promotion" or "behavior change" or "behaviour change") OR AB ("Health Promotion" or "wellness program" or "health promotion" or "behavior change" or "behaviour change") OR MW ("Health Promotion" or "wellness program" or "health promotion" or "behavior change" or "behaviour change")

100,768 results

### Workplace

TI ("Workplace" or "Employee*" or "worker*" or "office*" or "desk-based") OR AB ("Workplace" or "Employee*" or "worker*" or "office*" or "desk-based") OR MW ("Workplace" or "Employee*" or "worker*" or "office*" or "desk-based")

269,978 results

### Intersection of Health Promotion & Workplace

[S3] AND [S4]

8,967 results

Added to the Occupational Health search, we have:

[S2] OR ([S3] AND [S4])

1,312 results

## Concept 3: Eating and physical activity

TI ("Diet" or "Exercise" or "Sedentary" or "diet*" or "eating" or "exercise*" or "physical activiity") OR AB ("Diet" or "Exercise" or "Sedentary" or "diet*" or "eating" or "exercise*" or "physical activity") OR MW ("Diet" or "Exercise" or "Sedentary Behavior" or "diet*" or "eating" or "exercis*" or "physical fitness")

536,929 results

## Consolidated search

[S1] AND [S5] AND [S6]

TI ("behavior* change wheel" or "behaviour* change wheel" or "COM-B") OR AB ("behavior* change wheel" or "behaviour* change wheel" OR "COM-B") OR MW ("behavior* change wheel" or "behaviour* change wheel")

AND

(TI ("Occupational Health") OR AB ("Occupational Health") OR MW ("Occupational Health") OR (TI ("Health Promotion" or "wellness program" or "health promotion" or "behavior change" or "behaviour change") OR (AB ("Health Promotion" or "wellness program" or "health promotion" or "behavior change" or "behaviour change") OR MW ("Health Promotion" or "wellness program" or "health promotion" or "behavior change" or "behaviour change")) AND (TI ("Workplace" or "Employee*" or "worker*" or "office*" or "desk-based") OR AB ("Workplace" or "Employee*" or "worker*" or "office*" or "desk-based") OR MW ("Workplace" or "Employee*" or "worker*" or "office*" or "desk-based")) ))

AND

TI ("Diet" or "Exercise" or "Sedentary" or "diet*" or "eating" or "exercise*" or "physical fitness") OR AB ("Diet" or "Exercise" or "Sedentary Behavior" or "diet*" or "eating" or "exercis*" or "physical fitness") OR MW ("Diet" or "Exercise" or "Sedentary Behavior" or "diet*" or "eating" or "exercise*" or "physical activity")

56 results as of 2025-01-24 (up from 41 results in 2024).

# Nursing and Allied Health (ProQuest)

noft("behavior* change wheel" or "behaviour* change wheel" or "COM-B")

542 results

(noft("Health Promotion" or "wellness program" or "health promotion" or "behavior* change" or "behaviour* change") or SUBJECT("behavior modification")) AND noft("Workplace" or "Employee*" or "worker*" or "office*" or "desk-based")

8,399 results

noft("Diet" or "Exercise" or "Sedentary" or "diet*" or "eating" or "exercise*" or "physical fitness")

410,498 results

134 results

noft("behavior* change wheel" or "behaviour* change wheel" or "COM-B") AND (noft("Health Promotion" or "wellness program" or "health promotion" or "behavior* change" or "behaviour* change") or SUBJECT("behavior modification")) AND noft("Workplace" or "Employee*" or "worker*" or "office*" or "desk-based") AND noft("Diet" or "Exercise" or "Sedentary" or "diet*" or "eating" or "exercise*" or "physical fitness")

18 results as of 2025-01-24 (up from 15 results in 2024).

# Dissertations & Theses (ProQuest)

Repeat search used in Nursing and Allied Health:

6 results (no change in 2025)

# Google Scholar

"behavioural change wheel" "COM-B" workplace office eating physical fitness

[88 results](https://scholar.google.ca/scholar?start=0&q=%22behavioural+change+wheel%22+%22COM-B%22+workplace+office+eating+physical+fitness&hl=en&as_sdt=0,5&inst=8192625893198780794) as of 2025-01-24 (was 74 in 2024)

# **Updated Search (June 2026)**

The search was updated on June 16, 2026, using the same search strategy as the original search, with results limited to records added since January 2025. The updated search retrieved the following results:

| **Database** | **January 2025** | **June 2026** |
| --- | --- | --- |
| PubMed | 42 | 56 |
| EBM Reviews Multifile (OVID) | 11 | 16 |
| CINAHL | 56 | 59 |
| Nursing and Allied Health (ProQuest) | 18 | 26 |
| Dissertations & Theses (ProQuest) | 6 | 6 |
| Google Scholar* | 88 | 137 |

*Google Scholar returned 1,780 results in total, likely due to a change in their search algorithm. As Google Scholar does not allow retrieval beyond the first 200 results, the first 150 citations were downloaded and narrowed to 49 items published in 2025–2026.

In total, 66 unique records were identified. These were screened against the same eligibility criteria as the original search, resulting in the inclusion of 4 additional publications in the review.
